# Cancer Patients’ Social Relationships During 3 Years After Diagnosis—Generic and Cancer-Specific Social Networks

**DOI:** 10.1007/s12529-024-10292-4

**Published:** 2024-06-25

**Authors:** Ulla-Sisko Lehto, Markku Ojanen, Silja Lääperi, Mira Kohonen, Tommi Härkänen, Kirsi Honkalampi, Taina Turpeenniemi-Hujanen

**Affiliations:** 1 Population Health Unit, Finnish Institute for Health and Welfare (THL), Mannerheimintie 166, PO B.O.X. 30, FI00271 Helsinki, Finland; 2 Faculty of Social Sciences: Welfare Sciences, Psychology, University of Tampere, Tampere, Finland; 3 School of Educational Sciences and Psychology, University of Eastern Finland (UEF), Joensuu, Finland; 4 Cancer Center, Oulu University Hospital (OYS), Oulu, Finland; 5 Translational Medicine Research Unit, University of Oulu, Oulu, Finland

**Keywords:** Cancer patients, Cohort study, Follow-up study, Patient-reported, Methodology, Social networks, Social relationships

## Abstract

**Background:**

Social relationships are important health resources and may be investigated as social networks. We measured cancer patients’ social subnetworks divided into generic social networks (people known to the patients) and disease-specific social networks (the persons talked to about the cancer) during 3 years after diagnosis.

**Method:**

Newly diagnosed patients with localized breast cancer (*n* = 222), lymphoma (*n* = 102), and prostate cancer (*n* = 141) completed a questionnaire on their social subnetworks at 2–5 months after diagnosis and 9, 18, and 36 months thereafter. Generic and cancer-specific numbers of persons of spouse/partner; other family; close relatives, in detail; and friends were recorded as well as cancer-specific numbers of persons in acquaintances; others with cancer; work community; healthcare professionals; and religious, hobby, and civic participation. The data was analyzed with regression models.

**Results:**

At study entry, most patients had a spouse/partner, all had close relatives (the younger, more often parents; and the older, more often adult children with families) and most also friends. The cancer was typically discussed with them, and often with acquaintances and other patients (74–86%). Only minor usually decreasing time trends were seen. However, the numbers of distant relatives and friends were found to strongly increase by the 9-month evaluation (*P* < 0.001).

**Conclusion:**

Cancer patients have multiple social relationships and usually talk to them about their cancer soon after diagnosis. Most temporal changes are due to the natural course of life cycle. The cancer widened the patients’ social networks by including other patients and healthcare professionals and by an increased number of relatives and friends.

## Introduction

Social relationships have been investigated as *social networks* and *social support* [1–6], and from the 1990s onwards as *social capital* [7–10]. Social networks result in social support and social capital, but social networks as such also act as social health resources [1–3, 9]. The concept was first used by the anthropologists Barnes and Bott in the 1950s [11], who defined that a social network is a social structure made of nodes which are individuals or organizations. Later, a social network has been defined to be the web of social relationships surrounding an individual [6], or as “a specific set of linkages among a defined set of persons, … the characteristics of these … may be used to interpret the social behavior of the persons involved” [2]. Social networks describe the degree of social integration of a person in the social environment [2, 3, 12, 13]. Social networks comprise social ties between individuals [6] used for sharing, e.g., information, experiences, and support.

From the 1960s onwards, social networks have been found to be associated with lower mortality, better prognoses in chronic illnesses, and well-being [1–3, 13–18]. In the 1970s and 1980s, a series of population-based follow-up studies demonstrated that few or no social relationships predicted overall mortality [3, 19–22]. More abundant and diverse personal social networks were shown to predict a lower incidence and progression of various diseases and better well-being, whereas a lack of social networks and social isolation predicted poorer health [2, 3, 13–18, 23, 24]. Specifically, the size and diversity of a person’s social network are associated with health and longevity [15, 16, 18]. Fewer social ties and social networks measured by marital status, numbers of close relatives and friends, and membership in religious and voluntary associations have been found to predict mortality from almost all causes of death [2, 3, 6, 14, 23–25]. A causal impact on health has been shown [3], but the mechanisms through which social relationships affect health are not clear and multiple possible pathways have been suggested [6, 34].

Social networks are associated with health and well-being of cancer patients: there are many studies showing an impact on cancer progression, survival, and mortality [13–15, 26–31], and well-being of the patients [32, 33]. In the early 1990s, social network measures were shown to be predictors of both cause-specific and all-cause mortality among persons with a cancer disease [14]. In large population samples, social isolation (lack of interaction and participation) was found to be associated with an increased risk of cancer mortality [26, 27]. In 2010, a meta-analysis showed that large social networks are associated with longevity in cancer patients [15], and estimated that a larger social network is associated with a reduction of the relative mortality risk by 20%. Stronger associations of network size were observed in studies of breast cancer patients. It is noteworthy that most research in the area has investigated breast cancer patients. In addition, social relationships have been found to have both adverse and beneficial influences on breast cancer survival [27, 28], and the association between social networks and improved survival has been proposed to depend on the quality and function of the close relationships [28, 29, 34, 35]. The impact may depend on how burdensome a close relationship is to the patient, due to either the psycho-emotional burden they place on the patient and/or whether the patient takes care, helps, or acts as a caregiver for the loved one. Therefore, it would be essential to investigate cancer patients’ social networks with sufficient accuracy, especially so that the closest relationships can be separated from weaker social ties.

Social networks are the source of social support and the structural basis of social capital. In the middle of the 1970s, the study of social relationships and physical health was revitalized by the concept of social support [4, 5]. Many studies investigated whether the association was due more to the social support processes—protecting persons from effects of stressful events—than to the support structure, i.e., social networks [1–3]. The idea of social support that enhances adaptive behaviors or neuroendocrine responses in health-threatening situations quickly became the leading theory in health research [1–3, 6, 24, 36, 37]. When the concept of social capital emerged in research in the early 1990s, social structural aspects were re-emphasized. As social resources were found to be important for well-being [7, 9, 10, 38–40], the concept of social capital was defined to comprise some aspects of a social structure facilitating certain actions of individuals within the structure [9, 41, 42]. Social networks and participation are the structural components of social capital [8, 9, 12, 40–45].

Social subnetworks can be divided according to strength (weak/strong), direction (horizontal/vertical), formality (formal/informal), and diversity (bridging, bonding, and linking) [12, 42]. The most familiar is a classification into strong and weak ties, which may have different kinds of impact on health [8, 9, 12, 17, 23, 32, 34, 46]. Strong ties are typically those among family and friends and tend to be multi-stranded and regularly maintained, and they are often referred to as close networks. Weak ties are single-stranded and infrequently maintained, such as contacts with acquaintances. Formal contacts are based on organized activity, e.g., contacts between citizens and civil servants, while informal contacts usually take place among the strong ties. Formal and vertical ties include contacts with professionals, e.g., healthcare personnel treating a patient’s cancer.

In social support research, strong and informal networks are often seen as the most important ones because they sustain social relationships and provide sources of emotional support [1, 6, 13]. However, as stated above, close relationships not only can offer value and benefits but may also have adverse influences and cause stress [13, 28, 29, 32, 47]. In social capital research, weak and formal social networking and participation are seen to be of primary importance [7, 10, 40, 48] because they provide access to informational and formal instrumental support. Having many weak ties has been suggested to be able to compensate for the hazardous effect of a small number of strong ties on mortality [16]. Furthermore, especially the weak ties may facilitate well-being [17, 46].

Social networks can be measured by size (number of members), density (extent to which members know and associate with one another), boundedness (degree to which they are defined by traditional group structures), diversity (the number of different types of ties in a person’s network), and homogeneity (extent of similarity to each other) [6, 34]. Individual ties may be assessed as frequency of contacts, multiplexity (number of types of transactions/overlap in relationships), duration (length of time known), reciprocity (extent of transactions being reciprocal), and degree (quantifies the connections an individual has in a network). In health research, marital status, network size as number of confidants in total and/or frequency of contacts, overall connectedness, and participation in organizational activities are among the most common indicators used. The presence of social ties may be more important than the frequency of contacts [23, 49]. Networks can also be evaluated separately by different types of ties, i.e., by subnetworks [32, 50, 51], for example, by using a list of types of social relationship and measuring the number of people in each of them [52]. Measuring partial and purposive networks is based on lists of specific people who fill specific roles or perform special action in the respondents’ lives.

In our theoretical model [42], social networks in people with a chronic disease are divided into two types. First, *generic social networks* comprise people known to them, i.e., persons who are present in the lives of patients already before the diagnosis. Second, we suggest a new concept of *disease-specific social networks* emerging after the diagnosis and including person contacts related to the disease. A disease-specific social (sub)network is a purposive and partial network including the social ties to persons with whom the patient has discussed the disease [50]. It also comprises those involved in the treatment and care (such as healthcare personnel and support group members). Consequently, a disease-specific social network also includes the ties in the generic social networks with whom patients share information about the disease, at most as many persons as in the corresponding generic network. Receiving such medical treatment that includes person contacts widens a person’s social networks by the healthcare professionals involved. Cancer-specific social networks act as sources of cancer-specific social capital and cancer-specific social support [50, 53]. Both generic and disease-specific social networks are assumed to influence individual health and well-being, but possibly by different mechanisms.

Our aim was to evaluate the degrees of various aspects of social networks and social integration in cancer patients. We introduce the concept of cancer-specific social network and propose a method to measure (cancer) patients’ social networks as number of members in both generic and cancer-specific networks. In this study, cancer patients’ generic and cancer-specific social networks were measured during 3 years after diagnosis and in detail by different subnetworks. Also, temporal changes in the social subnetworks were investigated and their associations with background and clinical factors studied. We studied patients with the most common cancers in women and men, i.e., breast cancer and prostate cancer, which both have a good prognosis (5-year relative survival rate in Finland for breast cancer 92% and for prostate cancer 94%; www.cancerregistry.fi), and lymphoma(s), which are equally diagnosed in men and women and treated with various and often highly aggressive therapies with side effects.

## Methods

### Patients and Design

Newly diagnosed breast cancer (*n* = 225), lymphoma (*n* = 105), and prostate cancer (*n* = 150, with curative radiotherapy) patients with non-metastasized disease entering Oulu University Hospital (OYS) Department of Oncology, Finland, from May 2011 to May 2015 were consecutively enrolled in the study [51]. At 2–5 months after the diagnosis, the patients completed self-report questionnaires within an interview by a research nurse or researcher. Two breast cancer patients were afterwards excluded because they had a metastasized disease, three lymphoma patients because they had another type of cancer (chronic lymphatic leukemia) or a previous lymphoma diagnosis, and nine prostate cancer patients because they underwent radical prostatectomy prior to the radiotherapy. Also, the only male breast cancer patient was excluded from further analyses. The final study groups comprised 222 breast cancer, 102 lymphoma [48, women, 54 men], and 141 prostate cancer patients.

The patients completed questionnaires on their social networks, background and sociodemographic factors, other psychosocial factors, and well-being; only the social network measurement is reported here. The measurement was repeated by postal surveys at 9, 18, and 36 months after the interview. A completed follow-up questionnaire was returned by 91%, 85%, and 79% of the breast cancer patients; 84%, 72%, and 67% of the lymphoma patients; and 88%, 84%, and 77% of the prostate cancer patients (the non-response comprised non-participants and deaths; Table 1). The responding lymphoma patients were older (*P* = 0.045), but there was no difference in the follow-up participation by gender, education, other chronic diseases, and type of place of residence.

**Table 1:** Sociodemographic and clinical characteristics in patients (the hospital district is ethnically homogeneous regarding the age groups in question) at the study entry

	Breast cancer		Lymphoma		Prostate cancer	
	*n* = 222	(%)	*n* = 102	(%)	*n* = 141	(%)
*Gender*						
Man	–	–	54	(53)	141	(100)
Woman	222	(100)	48	(47)	–	–
*Age (years)*						
≤ 34	2	(1)	11	(11)	0	
35–44	15	(7)	9	(9)	0	
45–54	67	(30)	16	(16)	4	(3)
55–64	83	(37)	31	(30)	34	(24)
65–74	48	(22)	31	(30)	91	(65)
≥ 75	7	(3)	4	(4)	12	(9)
*Marital status*						
Never married	22	(10)	6	(6)	8	(6)
Married or cohabiting	165	(74)	83	(81)	113	(80)
Divorced or separated	25	(11)	8	(8)	15	(11)
Widowed	10	(4)	5	(5)	4	(3)
Missing	0		0		1	(1)
*Has children*	184	(83)	88	(86)	121	(86)
- Mean (SD)	2.3 (1.0)		2.9 (2.4)		2.5 (1.6)	
- Max	Max 6		Max 14		Max 13	
*Occupational education*						
No	19	(8)	12	(12)	42	(30)
Vocational couses	37	(17)	19	(19)	24	(17)
Vocational education/school	59	(27)	38	(37)	43	(31)
College	70	(32)	23	(22)	17	(12)
University education	36	(16)	10	(10)	15	(11)
Other or missing	2	(1)	0		0	
*Employment status*						
Employed	109	(49)	47	(46)	16	(11)
- Out of which part-time (< 30 h)	11	(10)	7	(15)	3	(2)
Unemployed	20	(9)	9	(9)	3	(2)
Retired	93	(42)	46	(45)	122	(86)
*Occupational status (current or pre-retirement)*						
Blue-collar	103	(46)	58	(57)	51	(36)
Lower white-collar	50	(23)	20	(20)	17	(12)
Upper white-collar	33	(15)	14	(14)	34	(24)
Self-employed, farmer, housewife	36	(16)	9	(9)	31	(22)
Other or missing	0		1	(1)	8	(6)
*Place of residence*						
City	102	(46)	48	(47)	58	(41)
Township	48	(22)	31	(30)	35	(25)
Rural area	72	(32)	23	(22)	48	(34)
*Other chronic disease/condition, number*						
0	84	(38)	51	(50)	36	(26)
1	85	(38)	30	(29)	44	(31)
2	38	(17)	17	(17)	34	(24)
≥ 3	14	(6)	4	(4)	27	(19)
- Hypertension	62	(28)	25	(24)	73	(52)
- Musculoskeletal disease	28	(13)	10	(10)	20	(14)
- Coronary heart disease	5	(2)	12	(12)	41	(29)
- Diabetes	3	(1)	10	(10)	29	(21)
- Chronic lung disease (asthma, COP)	9	(4)	7	(7)	17	(12)
*Grade*						
I	48	(22)	–	–	–	–
II	96	(43)	–	–	–	–
III	49	(22)	–	–	–	–
In situ	29	(13)	–	–	–	–
*Metastatic lymph nodes, number*						
0	164	(74)	–	–	–	–
1–3	36	(16)	–	–	–	–
> 4	22	(10)	–	–	–	–
Gleason classification *x* + *x*						
≤ 6	–	–	–	–	35	(25)
7–8	–	–	–	–	87	(62)
9–10	–	–	–	–	19	(14)
*Stage*						
I	–	–	31	(30)	–	–
II	–	–	9	(9)	–	–
III	–	–	24	(24)	–	–
IV	–	–	38	(37)	–	–
*Treatment*						
External beam radiotherapy	218	(98)	53^a^	(52)	141	(100)
Hormonal therapy	161	(73)	–	–	122^b^	(87)
Chemotherapy	89	(40)	90	(88)	–	–
- High dose (stem cell transplantation)	–	–	24	(24)	–	–
Rituximab	–	–	77	(76)	–	–
Lumpectomy	145	(65)	–	–	–	–
Mastectomy	83	(37)	–	–	–	–
*All-cause deaths during the follow-up*						
Before 9 months	0		6	(6)	0	
Between 9 and 18 months	0		2	(2)	1	(1)
Between 18 and 36 months	2	(1)	9	(9)	7	(5)

^a^Women received more often radiotherapy than men (*P* < 0.05)

^b^Neo-adjuvant

Clinical information on the cancer was collected from hospital records. Cancer care is centralized in Finland and OYS Catchment Area is the northernmost of the five university hospital districts in Finland.

### Study Questionnaire

To measure the patients’ social networks, an adapted version of the Structural–Functional Social Support Scale (SFSS) measurement tool [50, 54] was used. We adjusted the first part of the instrument (social networks) for the purposes of this study. First, the respondents gave according to a specific list the social subnetworks they had (presence of subnetworks), and second, they assessed the number of persons in each of the subnetworks (size of subnetworks) both as people known to them (generic networks) and as the number with whom they had talked about their cancer (cancer-specific networks). At study entry, the social network indicators were recorded since the diagnosis, and in the follow-up since the previous measurement.

The patients’ social networks were recorded as (1) spouse or intimate partner; (2) family members living in the same household (children < 18 years, children ≥ 18 years, other); (3) close relatives who did not live in the same household, but whom the patient had frequent contact with (adult children, spouses of adult children, grandchildren, own parents, spouse’s parents, own siblings, spouse’s siblings, other relatives); (4) close friends; (5) acquaintances they had talked to about the cancer; (6) other persons with a cancer diagnosis (any cancer, anytime); (7) doctors involved in examination and treatment of the cancer; (8) nurses involved in examination and treatment of the cancer; (9) work community members (co-workers, supervisors); and (10‒12) participation in a religious community, hobbies (max. three), and civic organizations (max. two).

Close subnetworks (categories 2–4, above) were measured as both generic and cancer-specific numbers of persons. In the more distant subnetworks (categories 5–9, from acquaintances to work community), only the cancer-specific number of persons was recorded, in acquaintances and in nurses with a classified variable. In participation (categories 10‒12), first the presence of the activity was recorded and next the number of social ties within the hobby or organization participation and the number of persons with whom the cancer was discussed.

### Statistical Analyses

The data were analyzed separately in the three cancer groups. Generic and cancer-specific social subnetworks were described as percentages (presence) and means and standard deviations (number of person) at the four measurement points. Temporal changes in the subnetworks were investigated by comparing the indicator at each measurement point with the corresponding one at study entry. A logistic regression model for the dichotomous outcome presence was used, and a population-averaged negative binomial regression model for the number of persons. These analyses were based on generalized estimating equations (GEE) and an exchangeable correlation structure. The models contained the measurement point as a categorical covariate. The associations of background and clinical factors with the social subnetworks over time were also estimated by these regression models, and in cancer-specific subnetworks adjusting for the corresponding generic subnetwork. A probability value (*P*) of ≤ 0.05 was considered statistically significant. Data were analyzed by IBM SPSS versions 24 and 25 (IBM Co., Armonk, NY) and STATA 16 (StataCorp LLC, College Station, TX, USA).

## Results

The breast cancer patients were on average 58 years old (SD 9.8, range 25–88), the lymphoma patients on average 56 years old (SD 14.1, range 20–80), and the prostate cancer patients on average 67 years old (SD 5.6, range 51–80). Thus, almost half of the breast cancer and lymphoma patients were in working life (Table 1), while the prostate cancer patients were mostly retired. Prostate cancer patients had a shorter education than the other groups (47% with no formal vocational education). Sixty-two percent of breast cancer patients, 50% of lymphoma patients, and 74% of prostate cancer patients reported another chronic condition.

Twenty-six percent of the breast cancer patients were diagnosed with lymph node metastases, 61% of the lymphoma patients were diagnosed with stage III–IV disease, and 14% of the prostate cancer patients had Gleason classification 9–10 cancer (Table 1). While most breast cancer and all prostate cancer patients were treated with external beam radiotherapy, lymphoma patients received multiple treatments (88% chemotherapy, 76% immunotherapy with anti-CD20 antibody rituximab).

### Social Networks

The presence and sizes of the patients’ generic and cancer-specific close networks (spouse, family/relatives, friends) are shown by subnetwork in Table 2. More distant networks, i.e., weak ties and formal ties (acquaintances, others with cancer, health care personnel, work community members), and participation are presented as cancer-specific numbers of persons by subnetwork in Table 3.

**Table 2:**
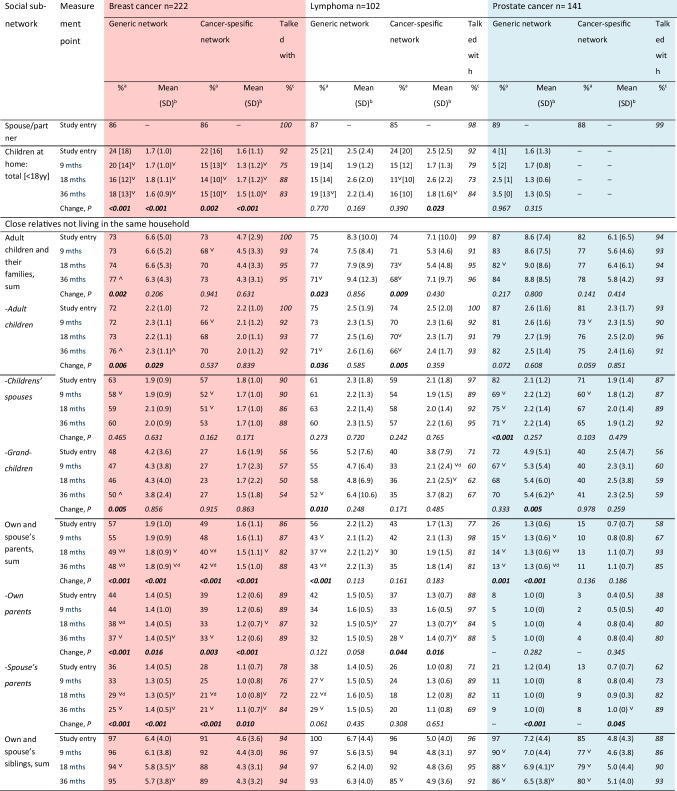

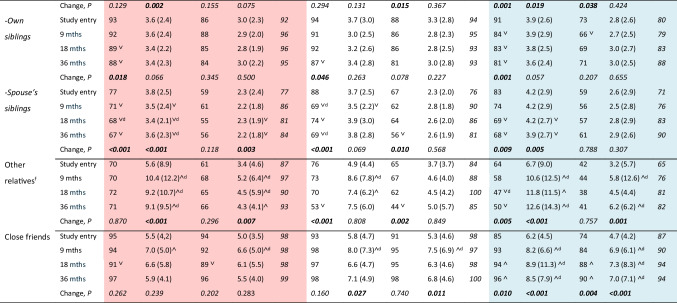
Patients’ generic and cancer-specific close social networks over the follow-up

Bold format indicates statistical significance *p*≤0.05

^∨^Statistically significant decrease in the temporal change analyses (all patients)

^∧^Statistically significant increase in the temporal change analyses (all patients)

^a^Presence of the subnetwork (no/yes)

^b^Average number of persons in a subnetwork, presented in those who have this network (omitting those without, i.e., the 0’s). *P*-value is based on the negative binomial regression model and the full data

^c^Proportion of persons in this subnetwork with whom the patients have discussed with about the cancer

^d^*P* ≤ 0.001

^e^Change over time between 9/18 and 36 months (reference class 9/18 months)

^f^In the follow-up, some patients answered “many,” which was classified as “10”

**Table 3:** Number of persons with whom the patients had talked about their cancer in their weak and formal social subnetworks and participation

Social subnetwork	Measurement point	Breast cancer	Lymphoma	Prostate cancer
		*n* = 222	*n* = 102	*N* = 141
		Mean (SD)^a^	Mean (SD)^a^	Mean (SD)^a^
Others with a cancer^b^	Study entry	2.7 (2.9)	2.6 (3.0)	3.5 (3.6)
	9 mths	3.3 (3.1)^˄^	3.4 (3.4)	3.8 (4.1)
	18 mths	3.2 (3.9)^˄^	4.8 (4.7)^˄^	3.9 (3.5)
	36 mths	3.8 (8.6)^˄^	4.9 (4.6)^˄^	4.5 (4.2)
	*Change over time, P*	**0.001**	**0.002**	0.150
Co-workers^c^	Study entry	8.8 (8.2)	6.4 (5.4)	6.1 (6.2)
	9 mths	9.1 (11.0)	5.6 (5.1)	8.1 (3.8)
	18 mths	9.8 (10.6)	4.3 (6.2)^˅^	10.4 (14.5)
	36 mths	6.3 (10.4)^˅^	3.6 (4.4)^˅^	5.5 (9.2)^˅^
	*Change over time, P*	**0.003**	**0.001**	** < 0.001**
Superiors	Study entry	1.2 (0.8)	1.2 (0.8)	0.9 (0.9)
	9 mths	1.0 (0.9)^˅^	1.3 (1.2)	1.4 (1.1)
	18 mths	1.1 (1.0)	0.8 (1.0)^˅^	2.3 (1.9)
	36 mths	0.9 (1.4)^˅^	0.7 (0.9)^˅^	0.7 (1.8)
	*Change over time, P*	**0.025**	**0.011**	–
Religious community	Study entry	10.4 (7.6)	7.7 (9.1) (*n* = 14)	5.0 (8.5) (*n* = 8)
	9 mths	7.4 (8.1)	5.1 (1.8) (*n* = 7)	5.2 (6.6) (*n* = 10)
	18 mths	9.8 (21.0)	14.3 (9.8) (*n* = 3)	5.0 (6.9) (*n* = 6)
	36 mths	4.6 (5.6)^˅^	10.8 (5.8) (*n* = 5)	8.0 (17.1) (*n* = 6)
	*Change over time, P*	**0.033**	0.737	0.208
Doctors treating the cancer	Study entry	5.0 (2.6)	5.3 (2.4)	3.4 (1.5)
	9 mths	1.6 (1.4)^˅^	3.2 (2.0)^˅^	1.2 (1.0)^˅^
	18 mths	1.5 (1.3)^˅^	2.9 (2.1)^˅^	1.0 (0.9)^˅^
	36 mths	1.7 (1.4)^˅^	2.3 (1.9)^˅^	1.2 (1.3)^˅^
	*Change over time, P*^d^	** < 0.001**	** < 0.001**	** < 0.001**
-	-	*Mode, class*	*Mode, class*	*Mode, class*
Nurses treating the cancer	Study entry	> 10	> 10	5–10
	9 mths	1–4	5–10	1–4
	18 mths	1–4	1–4	1–4
	36 mths	1–4	1–4	0
Hobby^e^	Study entry	1.9 (4.3)	2.3 (7.1)	1.5 (3.1)
Civic participation^e^	Study entry	5.4 (5.2)	4.9 (8.0)	1.6 (2.8)

Bold format indicates statistical significance *p*≤0.05

^∨^statistically significant decrease

^∧^statistically significant increase

^a^Average cancer-specific number of persons in each subnetwork in those who have this network

^b^Persons who had had any cancer diagnosis at any time

^c^Some unemployed and recently retired also reported these contacts

^d^Change over time between 9 and 36 months (reference class 9 months)

^e^Not measured in the follow-up

#### Strong Ties

Almost 90% had a spouse or intimate partner at study entry (Table 2). The mean duration of the partnership was 28 (SD 14.0) years in breast cancer, 31 (SD 14.9) years in lymphoma, and 36 (SD 14.7) years in prostate cancer patients. Slightly over half of the breast cancer and lymphoma patients lived in a two-person household with their spouse (54%, 59%) and one-quarter had children living at home (in all groups, typically 1 child), whereas in prostate cancer patients, living together with the spouse was more common (79%) and only few had children at home. Alone in a one-person household lived 20%, 14%, and 16% of the patients, respectively. In a few rare cases, there were also other persons in the household (1%, 3%, 1.5%), such as own or spouse’s parents.

Practically all spouses/partners were aware of the cancer at study entry. Exceptions were two lymphoma patients’ partners and one prostate cancer patient’s partner. Most patients had talked about the cancer with their home-living children (Table 2), but more seldom with children under 18 years of age.

All patients had at least some close relatives (not living in the same household but with frequent contacts: adult children, spouses of adult children, grandchildren, own parents, spouse’s parents, own siblings, spouse’s siblings, more distant relatives) at study entry (Table 2). The mean total numbers of close relatives were 16.1 in breast cancer patients, 17.9 in lymphoma patients, and 19.1 in prostate cancer patients. The most common subnetwork was siblings (own or spouse’s; present in 97–100%), and the patients had almost always talked with at least some of them about the cancer. Most often the cancer had been discussed with one’s own adult children. Three-quarters of the breast cancer and lymphoma patients had adult children with families in their close network, and at least some of them had talked about the cancer. More than half of these patients had own or spouse’s parents (57%), and 86% and 77% of them had talked about the cancer. However, almost nine out of ten prostate cancer patients (87%) had adult children with families (talked to 94%) and only one-fourth had parents (talked to 58%). Of female lymphoma patients, a larger proportion had own parents than of males (*P* = 0.02).

The more distant relatives were also often included in the close social network (64–76%) and the patients had often discussed the cancer with them, of the prostate cancer patients a smaller proportion than in the other cancer groups (87/84% vs. 65%) (Table 2). Most breast cancer and lymphoma patients (95% and 93%) and many prostate cancer patients (85%) had close friends, and practically all had discussed their breast cancer and their lymphoma with them, and the majority their prostate cancer (87%).

**Fig. 1:**
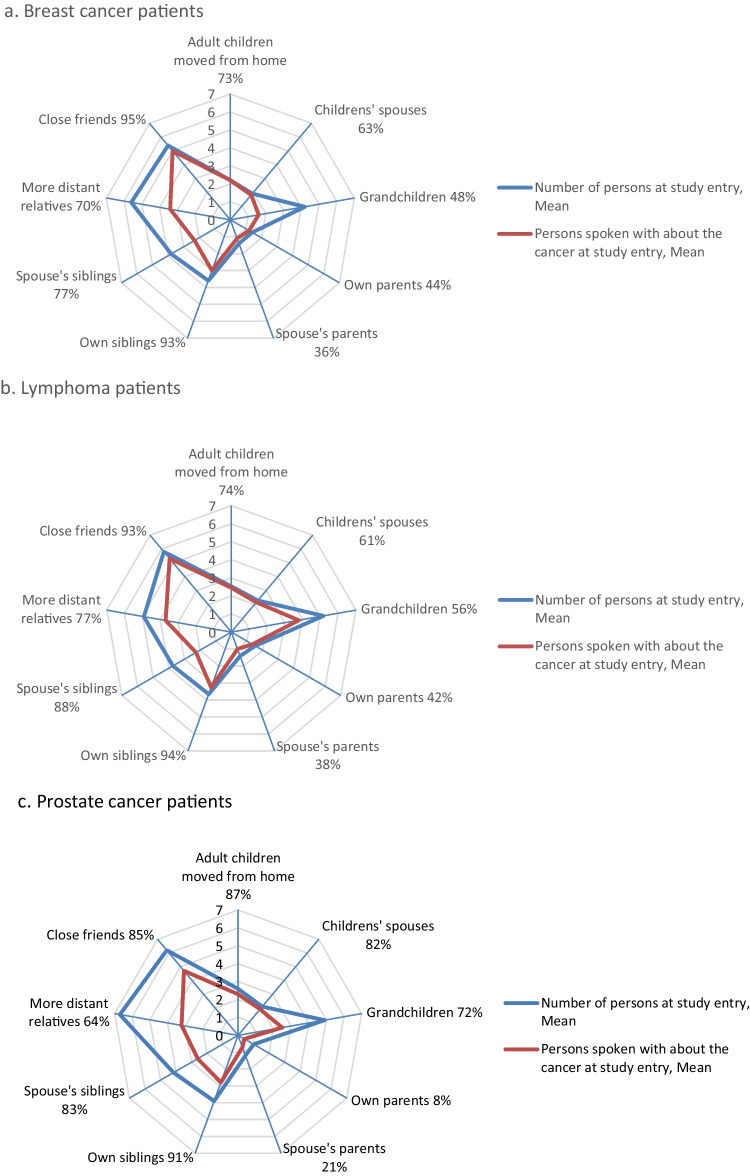
**a**–**c** Sizes of the generic and cancer-specific close subnetworks at the study entry (means in those with this subnetwork)

The sizes of the generic and cancer-specific close subnetworks at study entry are shown in Fig. 1. The closer the relatives were, the more similar were the sizes of the generic and cancer-specific subnetworks, i.e., the more often the cancer had been discussed, except with grandchildren. Prostate cancer patients had discussed with fewer of their strong social ties than other patients. Presence and number of persons in each of the close subnetworks at study entry did not differ between those participating in the follow-up and drop-outs/deaths.

#### Changes over Time in Strong Ties

Slightly fewer breast cancer patients had a spouse/partner at 36 months than at diagnosis (86 vs. 84%, *P* = 0.08), while there were only minor changes in the other cancer groups. At 36 months, a smaller proportion of breast cancer patients had children and fewer children living at home (Table 2). Also, breast cancer and lymphoma patients had fewer children under 18 years of age (*P*-values < 0.001 and 0.016). However, the presence and number of breast cancer patients’ adult children living elsewhere increased after 18 months and there were more often grandchildren, while in lymphoma patients, the presence of adult children and grandchildren decreased. In prostate cancer patients, the presence of adult children and their spouses decreased, and new grandchildren emerged. The presence and the numbers of parents (own and spouse’s) decreased substantially (*P-*values mostly ≤ 0.001).

Contrary to the commonly decreasing trend in the close subnetworks, the numbers of more distant relatives and close friends strongly increased between study entry and the 9-month evaluation in all cancer groups (*P*-values mostly < 0.001; Table 2). These numbers also did not decrease to the initial level during the follow-up. In prostate cancer patients, also the presence of close friends increased (*P* = 0.01).

#### Weak Ties

**Fig. 2:**
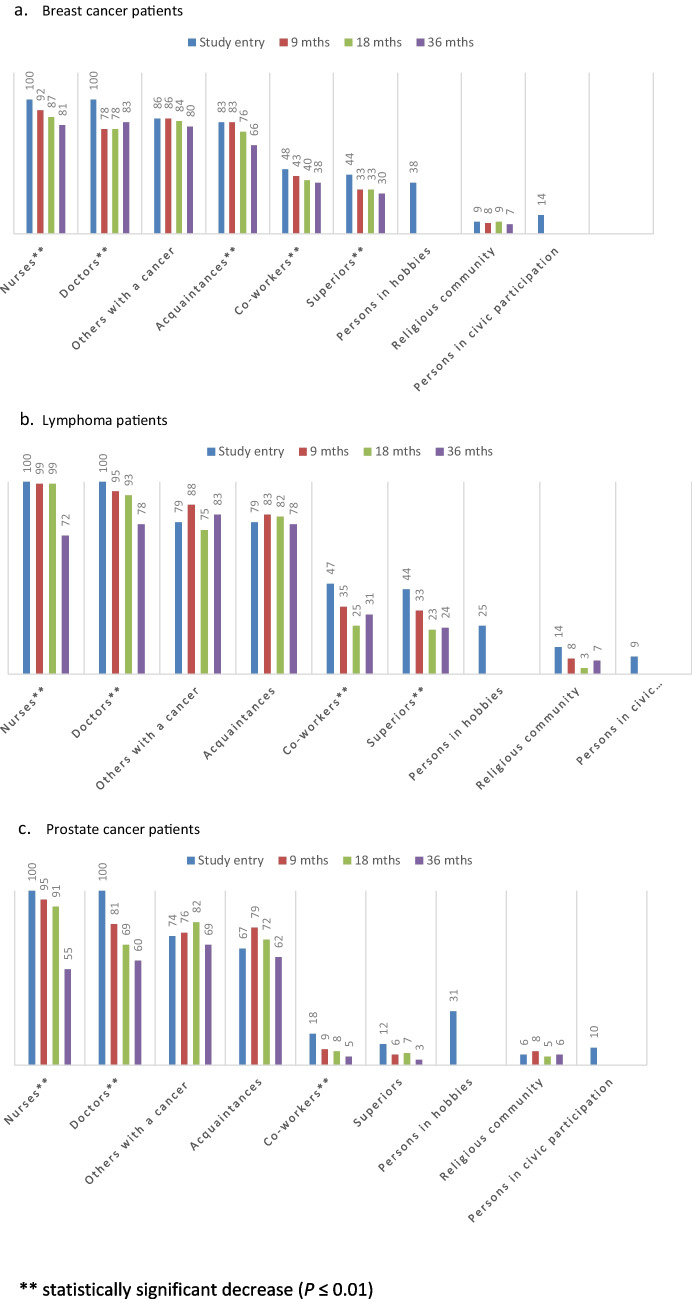
Presence (%) of weak ties with whom the patients had talked about the cancer over the follow-up. **a** Breast cancer patients. **b** Lymphoma patients. **c** Prostate cancer patients. **Statistically significant decrease (*P* ≤ 0.01)

By study entry, about 80% of breast cancer and lymphoma patients had discussed the cancer with acquaintances and other cancer patients (Fig. 2); in prostate cancer, the proportion reached the 80% level by 9 or 18 months. In breast cancer, talking to acquaintances became less frequent after 9 months (*P* < 0.001). Cancer patient support groups were rarely attended (by the study entry in 7%, 0%, and 2%, respectively). The numbers of other cancer patients talked to increased over the entire follow-up (Table 3). No gender differences were found in lymphoma patients. A larger number of other patients with whom the patients had discussed their cancer by study entry was associated with chemotherapy (in breast cancer, *P* < 0.01; in lymphoma, *P* < 0.08) and a high Gleason score (9‒10) in prostate cancer (*P* < 0.05).

The patients in working life (see Table 1) had already by study entry commonly discussed their cancer with their co-workers (87–94%) and supervisors (84–85%); breast cancer patients with on average nine co-workers (max. 45); and lymphoma and prostate cancer patients with more than six (max. 25 and 20) (Table 3). Information about the cancer diagnosis was typically shared with one supervisor. Cancer-specific work community contacts became rarer and fewer during the follow-up (Fig. 2, Table 3). More of the younger lymphoma patients talked about their cancer in their work community (*P* < 0.001).

#### Ties in Cancer Treatment

By study entry, on average, five doctors had contributed to the breast cancer or lymphoma diagnosis and treatment (ranges 1–20 and 2–12), and in prostate cancer 3.5 [1–10] (Table 3). One-fifth of the breast cancer and prostate cancer patients had not had any contact with a doctor/oncologist after the primary treatment, while in lymphoma patients this decrease took place only after 18 months (Fig. 2). At the beginning, usually more than 10 nurses had participated in examination and treatment; in prostate cancer patients 5–10.

#### Participation

At the beginning, 10–18% of the patients were active in a religious community, and 60–78% of them had talked about their cancer with on average 7.3, 7.7, and 8.8 persons (Table 3). Thus, 9%, 14%, and 6% of all patients had this cancer-specific subnetwork and these proportions did not change during the follow-up (Fig. 2). In breast cancer, the number of persons talked to decreased with time (down to 4.6, *P* = 0.002). Lymphoma patients’ religious community activity as such decreased from 18 to 12% (*P* = 0.01).

Although 71–84% of the patients had a hobby at study entry, and 50–65% had a hobby including personal contacts, only fewer than 40% (39%, 25%, 31%) of all patients had hobby contacts with whom they had discussed the cancer (Table 3), accounting for 74%, 38%, and 62% of the contacts.

Initially, 18% of breast cancer, 14% of lymphoma, and 24% of prostate cancer patients participated in civic organizations; the proportions were even larger among retired patients (*P* = 0.05). Although these activities included contacts with many persons (means 11–14), only about 10% (14%, 9%, 10%) of all patients had contacts with whom they had discussed their cancer (Fig. 2). Lymphoma patients’ participation in civic organizations increased up to 18 months (*P* < 0.05).

### Social Subnetworks and Background and Clinical Factors

Age was strongly associated with the presence of generic social subnetworks, while associations with other background factors were few. Older patients had more adult children, spouses of the adult children, and grandchildren in their inner circle, and there were fewer own and spouse’s parents (*P*-values ≤ 0.05‒0.001). No association between age and the number of friends was observed. Patients living in rural areas had more spouse’s siblings. The unemployed breast cancer patients had fewer distant relatives and fewer friends (*P*-values ≤ 0.05) and patients with other chronic diseases had fewer distant relatives (*P* < 0.01). There were more adult children and their families in the networks of lymphoma patients living in a township (*P* = 0.006) or in rural areas (*P* < 0.05), and retired patients had more distant relatives (*P* < 0.05). Prostate cancer patients living in a township (*P* = 0.01) or in a rural area (*P* = 0.001) had more distant relatives, and those with a short education had fewer distant relatives (*P* = 0.007).

After adjustment for the size of the generic social networks, older breast cancer patients talked about their cancer to a larger number of their children (*P* < 0.001) and all patients to a larger number of their grandchildren (*P*-values < 0.01). The older breast cancer and lymphoma patients talked less often (no/yes) with their distant relatives (*P*-values < 0.05). Breast cancer patients talked with their distant relatives more often if they were retired, had no other chronic diseases, or received chemotherapy (*P*-values < 0.05). Talking to acquaintances was less common if the breast cancer patients were unemployed or retired (*P* < 0.05) and more common if they received chemotherapy (*P* < 0.001). While a smaller proportion of older breast cancer patients discussed their cancer with other patients (*P* < 0.05), their number was larger if the patient was retired (*P* < 0.05) or had received chemotherapy (*P* < 0.001; in lymphoma patients, received stem cell transplantation, *P* < 0.05). Lymphoma patients living in rural areas talked to a larger number of their grandchildren (*P* = 0.007) and with a larger proportion of acquaintances (*P* = 0.003). Prostate cancer patients with a more severe cancer (Gleason 9‒10) talked to a larger number of distant relatives (*P* < 0.05), those living in rural areas less often with other cancer patients, and those living in a township to fewer other patients (*P*-values < 0.05).

## Discussion

We quantitatively explored cancer patients’ social relationships since diagnosis and up to 3 years. Social networks were evaluated in detail as counts of members in different social subnetworks and participation as the patients’ generic social networks, which are present regardless of the disease, and cancer-specific social networks, which emerge due to the disease and with whom the patients had discussed their cancer. We hereby introduce the concept of cancer-specific social networks and propose a methodology for measuring (cancer) patients’ social networks. 

The patients mentioned various social relationships, and they had talked to these persons about their cancer. At study entry, most patients had a spouse or an intimate partner, all reported having close family members/relatives outside the household, and most also close friends. By 2–5 months after diagnosis, everybody had talked with at least some members in each of their close subnetworks, and slightly fewer with grandchildren (obviously because some grandchildren were very young). Many patients had discussed their cancer also with acquaintances and other cancer patients. Those in working life had talked in their work community. These findings indicate that the cancer was in no way a secret but quickly shared with many people. However, speaking about the cancer in religious or civic participation activities was not common. The results correspond with our previous findings that people quickly and widely tell about their cancer diagnosis in their close social networks [50]. Furthermore, a more burdening treatment (chemotherapy) was associated with an increased number of people spoken to about the cancer.

Belonging to different demographic and socioeconomic groups (age, gender, marital status, work life, socioeconomic status) is known to result in different kinds of social subnetworks [15, 47, 55], which lead to different kinds of social structures and health resources. In this study, the presence of various social networks varied by age, but few associations with other sociodemographic factors were found. The younger patients had more often children still living at home, and own or spouse’s parents present (alive or well enough), and the older ones had more often adult children with families living elsewhere, including grandchildren. Altogether cancer patients are older than the population on average. Breast cancer and lymphoma patients are on average 60 years of age at diagnosis and prostate cancer patients over 70 years. Thus, their social networks usually are those of middle-aged and elderly people. Probably because our patients were older than the general population, a larger proportion had at the beginning of the study a spouse/partner (86‒89% vs. 63%) and they lived more rarely alone (14‒20% vs. 29%) and more often in a two-person household with their spouse (54‒79% vs. 47.4%) than the adult population aged 18 + years in Finland on average (see [56]). There is no detailed information available on the social networks of people in Finland by the subnetworks and by age groups. The breast cancer and lymphoma patients were about the same age (on average 58 and 56 years); and thus, their social subnetworks were quite similar. They differed from prostate cancer patients, who were about 10 years older, had rarely children at home, and were mostly retired. Because cancer incidence increases mainly in elderly people (www.cancerregistry.fi), the social networks of cancer patients will increasingly resemble those of the elderly population.

During the follow-up, close social networks were relatively stable; although small, usually decreasing changes were seen. An exception to the general decreasing trend was the considerable increase in the numbers of distant relatives and close friends between the first and the 9-month evaluation. Of the weak ties, the numbers of other cancer patients increased, while speaking with members in the work community decreased. Overall, there were more decreases in breast cancer patients than in lymphoma and prostate cancer patients, suggesting that the closest networks were even more stable in patients with a more severe cancer and more burdening treatments. 

The cancer seemed to add distant relatives and friends into the patients’ networks because the numbers of these were increased after the first measurement. This happened by 1 year after the diagnosis and the numbers did not fall back to the initial level. The reason for this must have been the cancer. The cancer may either have strengthened previous relationships (we asked about “close friends”) or brought entirely new relationships into these subnetworks. This occurred only after the primary treatment phase, when the patient may have had more psychological resources to promote and maintain relationships outside one’s immediate close network. The cancer might have provided willingness and need to sustain supportive social relationships. This finding is favorable because the size and diversity of a person’s social network are associated with health and longevity [15, 16, 18], and especially the more distant close relationships have been found to predict well-being in cancer [16, 32, 53]. 

Certain time trends observed in the close subnetworks were obviously due to the natural course of life cycle. These included the increasing numbers of adult children and grandchildren (children moved from home and grandchildren were born) and the decreasing numbers of parents (some became very ill or died). Such life changes are common in the same age groups in which people mostly are diagnosed with cancer. In cancer care, it is important to remember that cancer and cancer recovery are usually added to the developmental tasks of aging, such as empty nest, retirement, birth of grandchildren, and loss of older relatives.

To quantify social ties is rather demanding, especially for a large sample of subjects over prolonged periods of time. Thus, data collection has usually been restricted to one point in time, omitting the process nature of human relationships. The strengths of our study are the detailed measurement of the social relationships and the long follow-up time. Taking time trends and fluctuations in social relationships into account, the networks were assessed over several years. The subnetworks evaluated were found to be common, indicating that the method identified important subnetworks. The methodology was capable of identifying various aspects of social networks in people with a severe disease or a chronic condition. A good response rate was achieved up to 3 years. After 3 years, the impact of cancer on life and on the social relationships is probably further reduced if the cancer does not relapse or advance. Our data was representative of people diagnosed with breast cancer, lymphoma, and prostate cancer in Northern Finland (Oulu University Hospital Catchment Area, population about 740,000).

There are probably cultural differences in social networks between countries, which hamper the generalizability of the results. There are also differences in cancer care. At the time of the data collection, no consistent standards of psychological care were available in the area and related finances were limited; only patients in special need received professional consultation. In our study, there was no comparison group of non-cancer patients or healthy controls. Although we have no data on whether the patients had different networks than those without cancer, we believe that the generic social networks evaluated at study entry were similar to networks in the population of the same age. It is unlikely that the cancer had affected the existing (close) social relationships already soon after diagnosis. Also, we were not able to study social subnetworks during the course/progression of the cancer because the cancer types studied had good prognoses and rarely progressed during the period studied. Close social networks and strong ties were evaluated in more detail than weak ties, due to the obvious difficulty in mapping all social connections of a person, e.g., number of acquaintances. No analyses of the outcomes of the social networks are available. Cross-cultural comparisons and using the measure in chronic conditions other than cancer would be interesting. Our measurement tool is applicable in other chronic diseases and conditions too. 

This study offers a social perspective on people with chronic diseases by describing social relationships and the social environment of people after cancer diagnosis. We introduce the concept of cancer-specific networks and describe a new way to measure both generic and cancer-specific networks. We explored these in three samples of cancer patients. The patients had many and wide social subnetworks, in which they usually had talked about the cancer. Although almost all had close networks, their features differed by age. The cancer seemed to have added persons to and widened the patients’ social networks: there was no notable decrease over time, ties with healthcare personnel and other patients emerged, and ties with distant relatives and close friends increased substantially. As social networks may act both as risk factors and as protective factors for cancer patients, i.e., have both positive and negative effects on health and well-being [13], further research is needed to translate epidemiological findings on social networks into clinical and community strategies and to improve outcomes. It would be particularly interesting to study health and well-being outcomes resulting from the social subnetworks. Kroenke et al. [30, 34] recommend that social networks should be taken into account in clinical work as cancer patients’ social health resources, and in developing care and psychosocial interventions. Future studies should also evaluate cultural differences in cancer patients’ social subnetworks and the social subnetworks in relation to cancer progression.

## Data Availability

Data supporting the findings of this study are available within the article and from the corresponding author upon reasonable request.
